# Treatment and Filling of Root Canals*Reprinted in part from *The Journal N. D. A.* for September.

**Published:** 1919-10

**Authors:** F. W. Gethrow


### TREATMENT AND FILLING OF ROOT CANALS *

BY F. W. GETHROW, D.D.S.

The equipment necessary for pulp removal, pulp treatment and root filling is very limited. The important consideration in this treatment is a thorough asepsis which means surgical cleanliness. This means caring for the teeth to be included in the rubber-dam field and the

* Reprinted in part from *The Journal N. D. A.* for September.

cleansing of the adjacent soft tissues. The use of antiseptics for this part of the operation is unnecessary. Before the pulp chamber is entered, the rubber-dam, properly adjusted, is essential. After the rubber-dam is placed, the field should be made sterile by the use of a good antiseptic, such as oil of cloves, Black's one, two, three remedy, or phenol and then followed by the use of alcohol. The opening into the tooth should be made sufficiently large to see readily and operate without obstruction. With a sterile field and a good-sized opening so that all parts of the pulp chamber are readily accessible, the removal of the pulp or the enlarging of the canal should be done with barbed broaches. If barbed broaches are used as they should be used (never twisting them), I can assure you that the breaking of a broach in a root canal is quite impossible. The exact detail of the method followed in this curettement process is quite difficult to describe. The first broach to enter the root canal should not extend in more than two millimeters. The size of this broach must depend upon the size of the canal we are entering and the withdrawing motion of this barbed broach results in the curettement or enlarging of that portion of the canal. This should then be followed by a barbed broach somewhat larger and this method continued until that particular portion of the canal is as large as necessary for future thorough treatment and successful root filling. The rest of the process should be continued in the same manner until we have reached the apex of the root.

The question frequently asked is, "How is one to know when he has reached the apex of the root?" There are several things of importance that will assist us in determining when we have reached this point. The most important is to know that the normal foramina at root ends is much constricted and if we use broaches of proper size which will not penetrate these small foramina, we can, in most cases, measure very accurately the length of each root. Here the X-ray film is of great value, if it has been properly made, and we can measure the length of these roots on the films very accurately with a millimeter gauge. The element of pain indicated by the patient when a broach or other material comes in contact with the tissues beyond the end of the root is not at all reliable and should seldom be considered of value in determining this point. Nothing of any nature—broach, medicine, root canal pluggers or root fillings should ever penetrate through the end of the root. It should be remembered that there is the greatest difference in barbed broaches. Most barbed broaches are not sufficiently tempered to be effective cutting instruments for the curettement process. A barbed broach that is pliable and will bend and twist without breaking is practically useless for this process of curettement.

The correct interpretation of the X-ray film is a most important requisite. Various conditions, when taken from different. angles, may appear quite different or in many cases totally different. This could easily lead to a mistake in diagnosis which might lead to a very serious mistake in treatment. Many of the X-ray films are not well made and in most of these cases I consider it a mistake to attempt a diagnosis. We should invariably insist on a good clear film. We must be extremely careful about declaring that there could be nothing wrong with a case where the film seems to indicate a perfect condition. Many conditions such as dead pulp, acute abscessed conditions, etc., are rarely shown on an X-ray film. It is a serious mistake to attempt to make a diagnosis on the film alone except in some very marked conditions. The examination of the parts in conjunction with the film is essential. The average fees received for this class of operating is out of all proportion to the value and importance of the service. No discussion of this subject is complete without reference to this unfortunate condition. A surgeon can frequently perform one or more major surgical operations in the same time taken for the treatment of root canals of one tooth. There can be no doubt but that this particular point of poor fees is largely responsible for the poor results obtained in the treatment of teeth. The entire blame must be placed on the dental profession.

Assuming that we have root canals that are clean and sterile, I will now endeavor to cover the points to be followed in the filling of the canal. I can not believe there can be any serious discussion of the root filling material. Gutta-percha points are universally recognized as the very best material available. However, there is great variation in the material used in conjunction with the gutta-percha points. In my own practice, I use nothing but gutta-percha points and a sparing amount of eucalyptol.

The use of eucalyptol is essential in the filling of root canals for the following reasons:

First. It acts as a solvent for the gutta-percha.

Second. It acts as a lubricant for the gutta-percha points, greatly facilitating the root filling.

Third. It displaces any remaining moisture in the root canals because the eucalyptol has a great affinity for the dentin.

Fourth. It is an antiseptic.

There can be no reason for using anything excepting eucalyptol with gutta-percha in the larger canals. The great danger in the filling of the larger canals is that the material will penetrate the tissue beyond the end of the root. More than half of the root canals we are called upon to fill are in this class. If the other class—the small canals—are sufficiently enlarged they can be best filled by the same process. If these smaller canals are not enlarged to a reasonable size, chloropercha should be used preceding the placing of the gutta-percha points. The more chloropercha used, however, the poorer the operation. The length of the roots which has been established by the broaches and the X-ray films should be a vital part of the record kept of the treatment and filling of the root canals. A root canal plugger should be selected that will reach to within two millimeters of the end of the root. This measurement should be indicated on the root canal plugger so that the operator will known when this instrument has reached to within the two millimeter measurement of the apex. The diameter of the point of this plugger indicates the diameter of the gutta-percha point selected for filling. The gutta-percha point is picked up with a pair of pliers and cut to a length of about two millimeters. The end of the plugger point is slightly heated and attached to the larger end of the gutta-percha point. This eliminates the necessity of the operator's fingers coming in contact with any of the material entering the canals. The diameter of the gutta-percha point should be slightly in excess of the diameter of the root canal plugger point because the gutta-percha will pack and conform to the shape of the canal. If the root canal plugger with the gutta-percha point has been replaced in the canal to the measurement previously established, we are assured that this first piece of gutta-percha has reached the end of the root hermetically sealing the foramina. This first piece of gutta-percha spells success or failure in the filling process. I recommend that an X-ray film be taken with this first piece of gutta-percha in place. I can not think of any more exacting test of one's ability in root canal technic.

I know of no department in dentistry where operators are wasting as much effort and time as they are in the treatment of teeth.

I believe that 75 per cent. of the effort placed on the treatment of teeth is wasted.

I maintain that all teeth, regardless of conditions, can be successfully treated within a period of two or three days. Where a canal has been thoroughly curetted under aseptic conditions and when that patient returns the following day with such a canal, clean and sterile, that root is ready for root filling. A canal should be filled just as soon as it is clean and sterile. The sooner it is filled after reaching such a condition the better.

When we consider the conditions of the country at the present time and realize the importance of every one working at maximum efficiency, it should be the patriotic aim of every operator to eliminate all waste effort. Many operators throughout the country are wasting effort on teeth that can not possibly be returned to a healthy condition. It is well known that when the peridental membrane at the root ends has been destroyed and the cementum of these root ends are pus soaked that we can never hope to get a normal attachment of tissue to that portion of the cementum. No amount of treatment through to root canal will bring about a cure of such a condition.

There is one other point in the treatment of teeth that frequently escapes our attention. I want to refer very briefly to the very much impaired strength of the dentin as a result of the loss of the pulp. The loss of the pulp is one of the important steps in the loss of the tooth.

Practically every fracture of teeth is caused by the loss of the pulp. I believe that other conditions being equal, the loss of the pulp means a loss of at least 50 per cent. in the life of that tooth.

In this brief discussion I have tried to emphasize the following points:

1. The preservation of a healthy pulp is the most important service we can render.

2. When pulps have to be removed or teeth treated, surgical cleanliness is imperative. This means the same careful treatment for dead pulps, infected pulps abscessed conditions as for a normal healthy pulp.

3. The curettement process is the best and safest method for the removal of pulps and the enlargement of canals.

4. All small canals must be made sufficiently large for successful treatment and root filling.

5. No medicines must ever be used in the canal that could possibly injure the delicate tissues at the end of the roots.

6. No instrument of any nature should ever pass through the foramina.

7. Every treatment must be hermetically sealed. This eliminates all temporary stoppings, and many of the cements. Base plate gutta-percha is the best material for this purpose.

8. Make your operations thorough. Complete the treatment as quickly as possible. Most root canals can be filled within two or three days, regardless of complications.

9. Realize the value of your treatments and receive a fee worthy of this important service.

10. Remember that there is only one safe root filling and that is a normal healthy pulp.

The lesson to be learned from a study of this subject is that the removal of pulps and the subsequent treatment and root filling are to be looked upon as hazardous for the health of the patient. The success of these operations, even in the hands of the most skillful operators, is more or less uncertain.

We are not simply removing a nerve, we are removing tissue and in carrying out this process we are exposing our patients to all the dangers of surgical interference. We must realize that there are many difficulties encountered that will defy the skill of the best operator. Therefore pulp conservation is the keynote of this discussion.

